# Highlight signatures of vaginal microbiota and metabolome contributed to the occurrence and recurrence of vulvovaginal candidiasis

**DOI:** 10.1128/spectrum.01521-24

**Published:** 2024-10-30

**Authors:** Yiheng Liang, Zhuoqi Huang, Shangrong Fan, Changzhong Li, Liting Huang, Chunhua Huang, Andrew P. Hutchins, Chao Fang, Xiaowei Zhang

**Affiliations:** 1 Center of Obstetrics and Gynecology, Peking University Shenzhen Hospital, Shenzhen, China; 2 Shenzhen Key Laboratory on Technology for Early Diagnosis of Major, Gynaecological Disease, Shenzhen, China; 3 Institute of Obstetrics and Gynecology, Shenzhen PKU-HKUST Medical Center, Shenzhen, China; 4 Department of Systems Biology, School of Life Sciences, Southern University of Science and Technology, Shenzhen, China; 5 Laboratory of Genomics and Molecular Biomedicine, Department of Biology, University of Copenhagen, Copenhagen, Denmark; Universita degli Studi di Modena e Reggio Emilia, Modena, Italy

**Keywords:** recurrent vulvovaginal candidiasis, *Lactobacillus iners*, *Prevotella bivia*, glycogen metabolism, L-glutamate

## Abstract

Vulvovaginal candidiasis (VVC) is a common vaginal infectious disease caused by *Candida*. The high recurrence rate of VVC is a great clinical challenge, with recurrent VVC (RVVC) defined as four or more episodes within a year. In this study, we recruited 31 RVVC patients, 28 VVC patients, and 29 healthy women. Vaginal samples were collected for metagenomic and metabolic analysis. RVVC and VVC groups presented similar clinical symptoms, with only a significantly increased incidence of swelling in the VVC group. Vaginal microbiota in VVC/RVVC exhibited a decreased abundance of *Lactobacillus* and increased bacterial vaginosis-associated bacteria, such as *Gardnerella*, *Prevotella*, and *Atopobium*. Notably, *Lactobacillus iners* was higher in RVVC, suggesting not all *Lactobacillus* species are protective. Healthy women showed lower overall microbiota diversity, emphasizing single-species dominance for stability. Glycogen metabolism pathways were enriched in RVVC/VVC, and were correlated with *Atopobium vaginae*, *Prevotella bivia*, and *Lactobacillus jensenii*. Peptidoglycan synthesis pathways, associated with *P. bivia*, were enriched, with the substrate L-glutamate elevated in RVVC, possibly promoted by *L. iners*. These findings shed light on potential therapeutic targets for recurrent VVC, contributing to the understanding of the intricate interplay between the metabolism of vaginal microbiome and disease.

**IMPORTANCE:**

This study enhances our knowledge of the vaginal microbiota dynamics and the role of associated metabolites in individuals with vulvovaginal candidiasis (VVC) and recurrent vulvovaginal candidiasis through shotgun sequencing and multi-omics analysis. The relationship between metabolites and vaginal microbiota and disease state was revealed. The accumulation of L-glutamate generated in glycogen metabolism, which is governed by *Lactobacillus iners* or bacterial vaginosis-associated bacteria, may contribute to the incidence and recurrence of VVC. Such insights have the potential to impact the treatment and prevention strategies for these common yet distressing conditions, potentially leading to targeted therapies and improved patient outcomes.

## INTRODUCTION

Vulvovaginal candidiasis (VVC) is the most prevalent human *Candida* infection, estimated to afflict approximately 75% of all women at least once in their lifetime. Moreover, recurrent VVC (RVVC, defined as >3 episodes per year) affects nearly 8% of women globally (1). Even after treatment, the complete cure rate for RVVC is less than 50% (2), which further exacerbates life burden and affects the life quality of patients. The occurrence of VVC is regulated by multiple factors, including host, pathogen, and vaginal environment. The vaginal environment includes vaginal pH, sex hormones, microbiome structure, and sexual activity (3). Among these factors, vaginal microbiota plays a significant role in the development of vaginal infections (4). Ravel et al. identified five vaginal microbial community state types (CSTs), of which four have *Lactobacillus* as the predominant organism, including CST I, CST II, CST III, and CST V, which were dominated by *Lactobacillus crispatus*, *Lactobacillus gasseri*, *Lactobacillus iners*, and *Lactobacillus jensenii*, respectively. While CST IV is characterized by the absence of Lactobacillus spp., it is usually dominated by a variety of facultative anaerobes, such as *Gardnerella, Prevotella*, and *Megacoccus* (5, 6). Despite this different microbial composition, CST IV can still represent as a balanced environment in healthy women. *Lactobacillus* plays a crucial role in maintaining the acidic environment of the vagina by producing lactic acid and creating an anaerobic environment through the production of H_2_O_2_. This helps in preventing invasive pathogens (7, 8). The reduction of *Lactobacillus*, which may cause dysfunction of the vaginal microbiota, will result in an inability to protect against infections, leading to various vaginal infections, such as bacterial vaginosis (BV) and aerobic vaginitis (AV) (9). However, in contrast to BV and AV, VVC is a fungal infection that exhibits a more complex interaction between *Candida* and the vaginal microbiota. Some studies have suggested that microbial dysbiosis, including the decrease in Lactobacillus and increase in facultative anaerobes, is linked with *Candida* infection (10, 11), which may be due to the enhanced inflammatory response. Reduced levels of *Lactobacillus* lead to the production of insufficient lactic acid, which activates pro-inflammatory mediators by releasing lower amounts of the anti-inflammatory cytokine IL-1RA (12). Meanwhile, the overgrowth of facultative anaerobes, such as *Gardnerella vaginalis* and *Prevotella amnii*, induces the production of pro-inflammatory interleukins, including IL-1β and IL-12 (13). However, other research has indicated that the abundance of *Lactobacillus* genus does not decrease in VVC patients (14). Similar findings have been documented in studies focusing on cases of RVVC. Traditional culture- or PCR-based studies have revealed that the *Lactobacillus* species were detected or dominated in the vaginal microbiota of women with RVVC, such as *L. crispatus*, *L. iners*, *L. gasseri, L. acidophilus*, and *L. fermentum* (15, 16). On the other hand, studies utilizing next-generation sequencing technology have suggested that the vaginal microbial composition in RVVC cases is comparable to or slightly more diverse than that of healthy individuals (13). In one study employing 16S rRNA sequencing to analyze RVVC patients, a significant reduction in health-associated *L. crispatus* and *L. jensenii* was observed. Conversely, patients exhibiting positive *Candida* cultures and those experiencing RVVC for over 6 months displayed a 21% increase in *L. iners* (17). The influence of bacteria on VVC recurrence appears to be uncertain and complex. More evidence is needed to establish the association between *Lactobacillus* abundance, vaginal microbiota, and the occurrence/recurrence of VVC. Moreover, further exploration of the role of vaginal microbiota in VVC/RVVC patients is warranted to investigate the intricate relationship between the vaginal environment and *Candida* infection.

Significant alterations in vaginal metabolite composition have also been observed in VVC patients. Decreased lactic acid is a common hallmark of all pathological conditions. The contents of TMA-Nox (TMAO), taurine, and methanol were increased in the VVC metabolic profile compared with the healthy group. The levels of isopropanol, O-acetylcholine, and glucose in the VVC metabolic profile were similar to those in BV, which all showed increases compared to healthy cases. In addition, serum lactic acid, 4-hydroxyphenylacetic acid, phenylalanine, pi-methylhistidine, and glycine concentrations were significantly lower in VVC and BV women. Both dimethylamine and sarcosine were significantly reduced in all pathological conditions compared with healthy subjects. The metabolite differences may ultimately result from vaginal microbiota (4). But currently, there are limited studies that examine the association between vaginal microbiota composition and metabolomics in VVC/RVVC patients.

The objective of this study is to investigate the compositional and metabolic characteristics of vaginal microbiota in patients with RVVC, VVC, and healthy individuals using metagenomic sequencing techniques. Additionally, metabolite profiles using high-performance liquid chromatography-mass spectrometry was performed to identify the contribution of bacteria to the vaginal metabolic environment. This study explores potential treatment targets of vaginal microbiota on the occurrence/recurrence of VVC.

## MATERIALS AND METHODS

### Collection of subjects and samples

The study was completed at Peking University Shenzhen Hospital, screening women with vulvar itching and white curd‐like discharge who visited the gynecology clinic of Peking University Shenzhen Hospital between January 2022 and December 2022. The inclusion criteria included participants who (i) are 18–52 years old that are or were sexually active, (ii) meet the diagnostic criteria for VVC and RVVC 4 or more episodes of symptomatic VVC within 1 year, gynecological examination of vaginal discharge, microscopic examination of fungal pseudohyphae and budding spores) or are healthy: vaginal cleanliness II degree, free of *Candida*, *Trichomonas*, aerobic bacteria, and bacterial vaginosis, and (iii) signed informed consent. Exclusion criteria were (i) women who are pregnant or breastfeeding or intend to become pregnant; (ii) long-term use of contraceptives, immunosuppressants; (iii) menopausal; (iv) patients with severe cardiac, liver, and renal function abnormalities, patients with psychiatric disorders, patients with infectious diseases, patients with tumors, patients with severe anemia, patients with severe autoimmune-type diseases combined (e.g., rheumatoid arthritis, lupus erythematosus, etc.), etc.; (v) patients with severe gastrointestinal diseases, including colorectal cancer, irritable bowel syndrome (IBS), inflammatory bowel disease (IBD), chronic or acute diarrhea, long-term constipation patients, etc.; (vi) patients who have received gastrointestinal surgery and abdominal surgical treatment patients within 1 year, such as cholecystectomy, etc.; (vii) menstruation period; (viii) sexual activity within 72 hours; (ix) any vaginal medications (such as vaginal douches, creams, or vaginal ovules) used within 48 hours prior to the test. All samples of vaginal discharge were collected by full-time clinical research physicians using swab (95000LV from MANTACC for vaginal discharge collection for metagenomic sequencing, KJ502-2 from Kangjian for vaginal discharge collection for metabolic sequencing).

### Culture and Identification of *Candida*

The swab samples were inoculated onto Sabouraud’s agar plates and then incubated at a constant temperature of 37°C for a period ranging from 24 to 72 hours, in an inverted position. Following incubation, well-grown single colonies of uniform morphology were selected and transferred onto CHROMagar *Candida* medium. These samples were again incubated in an inverted orientation at 37°C for 24 to 48 hours to facilitate observation of the chromogenic reactions. The resultant colony colorations were identified as follows: bright green indicated *Candida albicans*; gray-blue signified *Candida tropicalis*; pink denoted *Candida krusei*; purple was characteristic of *Candida glabrata*; and colonies with a purple center framed by white boundaries were indicative of *Candida parapsilosis*.

### DNA extraction and metagenomic sequencing

Bacterial DNA from vaginal discharge was extracted utilizing the MagPure Universal DNAKF Kit (1 mL per sample). The concentration of extracted DNA products was quantified using the Qubit dsDNA HS Assay Kit, and utilized agarose gel electrophoresis to verify the integrity of the DNA. All samples used in our analyses met stringent quality standards, with concentrations exceeding 8.0 ng/µL and total amounts greater than 0.2 µg. All samples were subsequently interrupted by a Covaris E220 non-contact ultrasonic crusher and purified by AxyPrep nucleic acid isolation kit. The DNA library was prepared using MGIEasy Fast FS DNA Library Prep Set. Metagenomic sequencing was performed on the DNBSEQ platform (BGI, Shenzhen, China) (100 bp paired end mode). Quality control of sequence reads was conducted using fastp (v.0.20.1), with default parameters. High-quality sequences were then aligned to the hg38 human reference gene set using Bowtie2 v.2.4.2 to remove the host genome sequences. HUMAnN v.3.0, utilizing a hierarchical algorithm, mapped sequences into species marker genes, pan-genome, and protein databases. This enabled taxonomic and functional annotation of the high-quality sequences after host removal. HUMAnN v.3.0 was used to obtain high-fidelity microbial taxonomic and metabolic profiles. In this study, the abundance of dominant species (ADS) was delineated as the proportion of the most abundant taxonomic entity within each microbiome sample, irrespective of the taxonomic identity of the dominant organism across different samples.

### Vaginal discharge metabolite extraction and liquid chromatography-tandem mass spectrometry (LC-MS) analysis

To prepare vaginal discharge metabolites, we added each swab sample to an Eppendorf tube containing 500 µL of 80% methanol, followed by vigorous vortexing and 15 minutes of sonication. The samples were then incubated at −20°C for 30 minutes to precipitate proteins. Then, we squeezed the swabs, centrifuged the samples at 20,000 rcf for 15 minutes at 4°C, and transferred 300 µL of the supernatant to a new tube. This was repeated, and 200 µL of supernatant was collected for UPLC-MS analysis (UltiMate 3000 UPLC, Thermo, USA). Additionally, a pooled sample consisting of 10 µL from each individual sample was prepared as QC (quality control) sample to ensure consistency and reliability across the analytical procedures. The entire extraction procedure was conducted on ice to preserve the integrity of the samples (18). The processing of the acquired MS data from each experimental sample and QC data generated from QC samples, including peak picking, peak grouping, retention time correction, second peak grouping, as well as annotation of isotopes and adducts, was performed using XCMS software. The LC-MS raw data files were converted into mzXML format and subsequently processed by XCMS, CAMERA, and metaX toolboxes within the R environment. Each ion was identified by its retention time and mass-to-charge (m/z) ratio, with the intensities of each peak being recorded. These steps resulted in the creation of a three-dimensional matrix, comprising arbitrarily assigned peak indices (pairs of retention time-m/z), sample names (observations), and ion intensity information (variables).

Further preprocessing of the intensity data for peaks was conducted using metaX, wherein features detected in less than 50% of the QC samples or 80% of biological samples were discarded. For the remaining peaks that contained missing values, imputation was performed using the k-nearest neighbor algorithm to enhance the data quality. The metabolomics data underwent normalization using the probabilistic quotient method. Quality control-based robust locally estimated scatterplot smoothing (LOESS) signal correction was applied to the QC data in accordance with the injection order to reduce signal intensity variations over time. In addition, relative standard deviations of the metabolic features were calculated across all QC samples, and those with values >50% were removed.

### Statistical analysis

Microbial α-diversity indexes, including the Shannon diversity and the Simpson diversity, were calculated using the vegan package. Continuous variables were compared using a two-sided Wilcoxon rank-sum test, while Fisher’s exact test was applied for the comparison of categorical variables. Correlations between taxa and metabolites were calculated using Spearman’s method. Supervised Partial Least Squares Discriminant Analysis (PLS-DA) was conducted via metaX to distinguish the different variables between groups. The variable importance in projection (VIP) value was calculated and a cut-off value of 1.0 was used to identify features of significance. Finally, results were visualized with a custom R script, primarily using ggplot2. All statistical analyses were performed using R version 4.3.

## RESULTS

### Clinical demographics and characteristics

**Fig 1:**
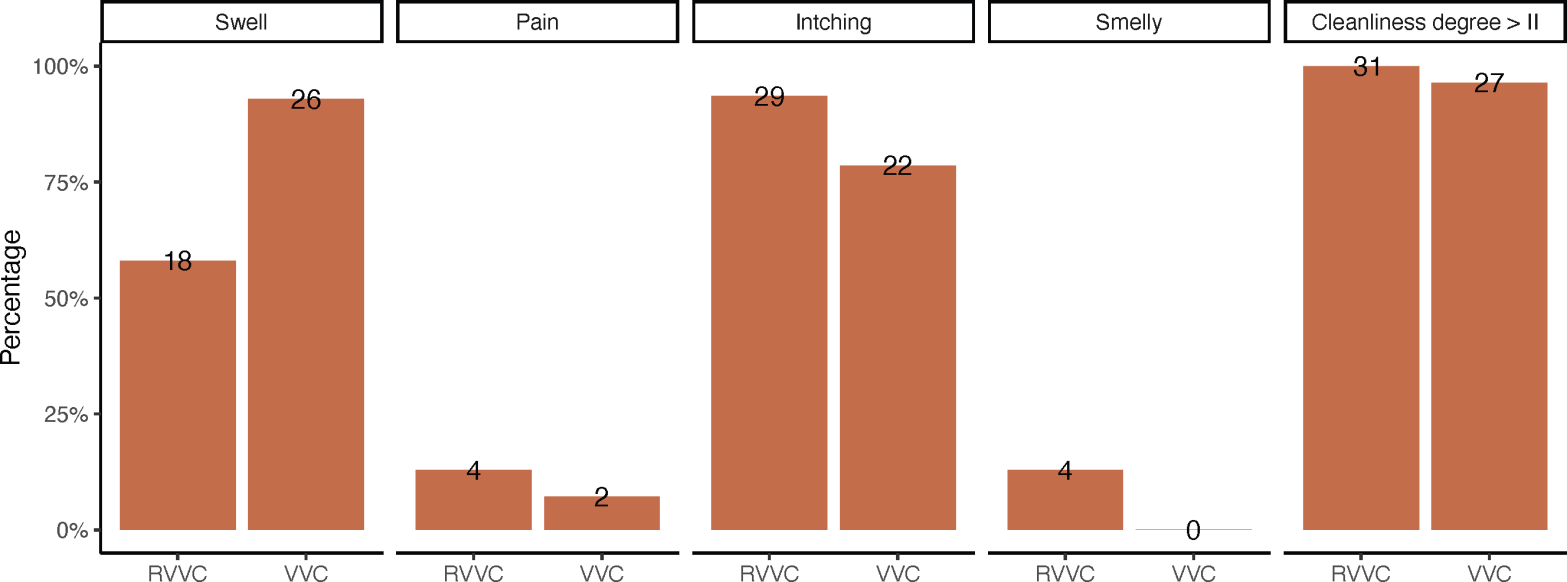
VVC is more likely to be associated with acute inflammation than RVVC. The percentages of clinical symptoms observed among the participants are graphically presented for both groups: 31 with RVVC and 28 with VVC. The numerical values displayed on the bars represent the participant count associated with each respective symptom. *P*-value was calculated by Fisher’s exact test.

A total of 94 participants were initially recruited, of which 87 were included in the analyses for this study. Thirty women were initially recruited for the healthy group, but one participant was found to be pregnant after enrollment and was therefore excluded, leaving 29 cases. Among the VVC patients, there were 30 cases, of which 2 were diagnosed with BV co-infection and were excluded, leaving 28 cases. Among the RVVC patients, there were 34 cases, of which 2 BV co-infection and 1 AV co-infection were excluded, leaving 31 cases. Clinical symptoms were collected for both VVC and RVVC patients, including odor, itching, burning, swelling of the external genitalia, and cleanliness degree of vaginal discharge (Fig. 1). After conducting Fisher’s test, it was found that the incidence of swelling was significantly higher in the VVC group compared to the RVVC group, suggesting that VVC is more likely associated with acute inflammation.

### Distinct differences in vaginal microbiota detected among the RVVC, VVC, and healthy groups

**Fig 2:**
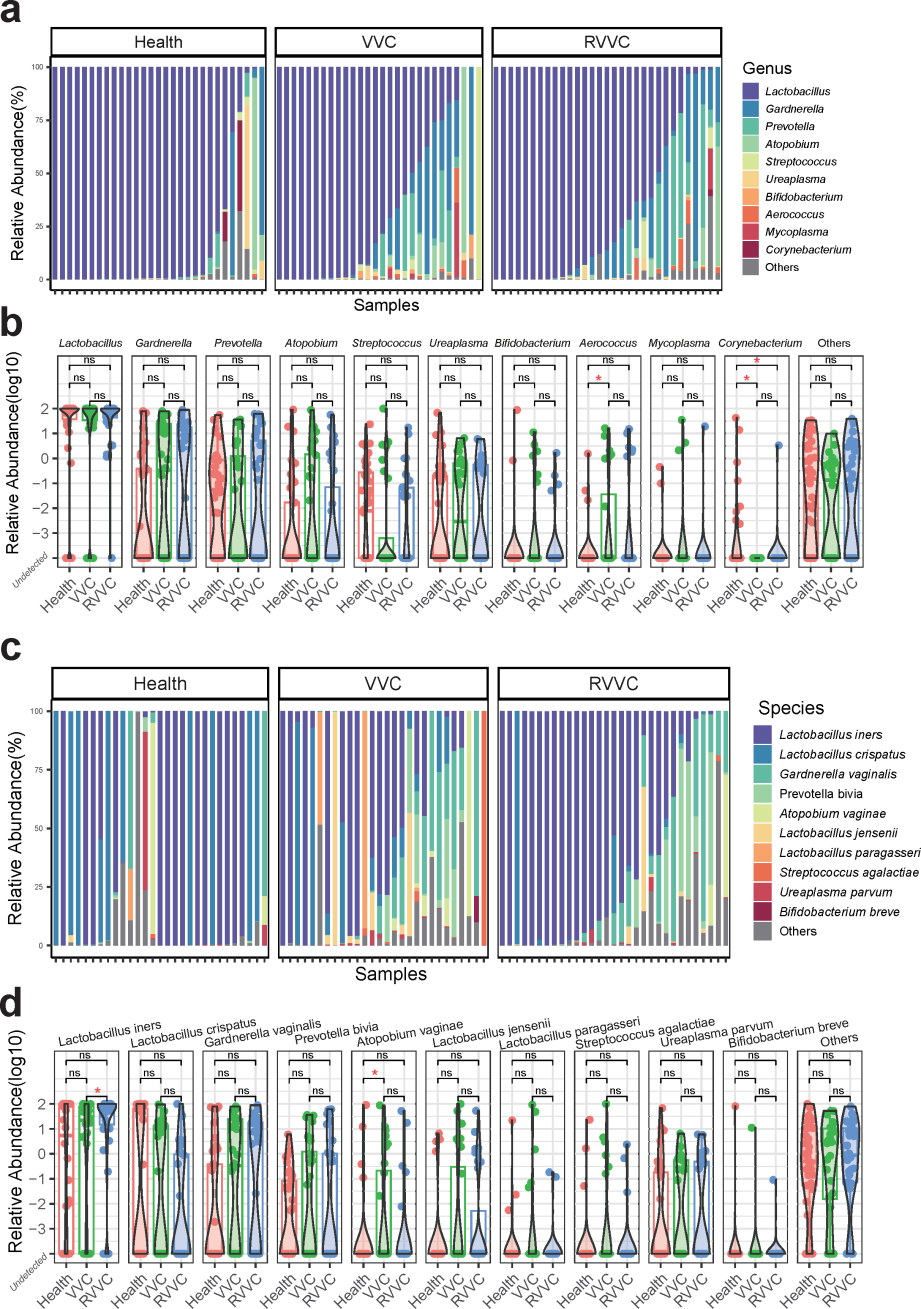
Taxonomic difference analysis between groups. (a) The proportion of the top 10 genus abundance of vaginal flora in each sample. Among them, the highest abundance is *Lactobacillus*, and other high abundance is mostly common BV pathogens, including *Gardnerella*, *Prevotella*, *Atopobium*, etc. (b) Differences among groups of the top 10 genus of vaginal flora. The results showed that the abundance of *Corynebacterium* was significantly increased in the healthy group and that of *Aerococcus* was significantly increased in the VVC group. (c) The proportion of the top 10 species abundance of vaginal flora in each sample. Among them, the highest abundance of *Lactobacillus iners* and *Lactobacillus crispatus*, and other high abundance common BV pathogens, including *Gardnerella vaginalis*, *Prevotella bivia*, *Atopobium vaginae*, and so on. (d) The abundance of *Atopobium vaginae* was significantly higher in VVC than in the healthy group, but there was no significant difference between the VVC and RVVC groups or between the RVVC group and the healthy group. The abundance of *Lactobacillus iners* was significantly higher in the RVVC group than in the VVC group, but there was no significant difference between the RVVC group and the healthy group or the VVC group and the healthy group. Statistical significance was calculated using Wilcoxon signed-rank test (red *, *P* < 0.05; ns, non-significant).

Metagenomic analysis of vaginal microbiota from the RVVC, VVC, and healthy groups revealed distinct differences in the abundance of genera among the three groups. *Lactobacillus* was the most abundant genus in all three groups, comprising 69.56% in average of the total composition (73.11% in healthy group, 66.28% in VVC group, and 68.17% in RVVC group). Other genera in the top 10 by abundance included *Gardnerella* (11.32%), *Prevotella* (5.93%), *Atopobium* (3.70%), *Streptococcus* (1.88%), *Ureaplasma* (1.34%), *Bifidobacterium* (1.13%), *Aerococcus* (0.80%), *Mycoplasma* (0.63%), and *Corynebacterium* (0.62%) (Fig. 2a). A comparative analysis between the groups demonstrated an increasing trend in the common BV-associated pathogens *Gardnerella*, *Prevotella*, and *Atopobium* in the VVC and RVVC groups, although these differences were not statistically significant. The abundance of *Corynebacterium* was significantly higher in the healthy group compared to the RVVC and VVC groups, with no significant difference observed between the RVVC and VVC groups. Additionally, the abundance of *Aerococcus* was significantly higher in the VVC group compared to the healthy group, but no significant difference was observed between the RVVC group and either the healthy or VVC group (Fig. 2b).

Furthermore, species-level analysis of vaginal microbiota between groups showed that the most abundant species observed in all three groups was *Lactobacillus iners*, accounting for 47.16% in average of the composition. Other abundant species included *Lactobacillus crispatus* (16.43%), *Gardnerella vaginalis* (11.32%), *Prevotella bivia* (4.43%), *Atopobium vaginae* (3.0%), *Lactobacillus jensenii* (2.7%), *Lactobacillus paragasseri* (1.69%), *Streptococcus agalactiae* (1.41%), *Ureaplasma parvum* (1.29%), *Bifidobacterium breve* (0.94%), and others comprising 9.70% (Fig. 2c), and the relatively abundance of each species were varied among three groups (Table S1). Comparative analysis at the species level revealed a significant increase in the abundance of *A. vaginae* in the VVC group. Additionally, the abundance of *L. iners* showed a significantly higher level in the RVVC group, indicating its association with recurrence. However, no significant differences were observed between the RVVC group and either the healthy or VVC group (Fig. 2d).

### No significant association between the *Candida* species and the vaginal microbiota in the RVVC and VVC groups

**Fig 3:**
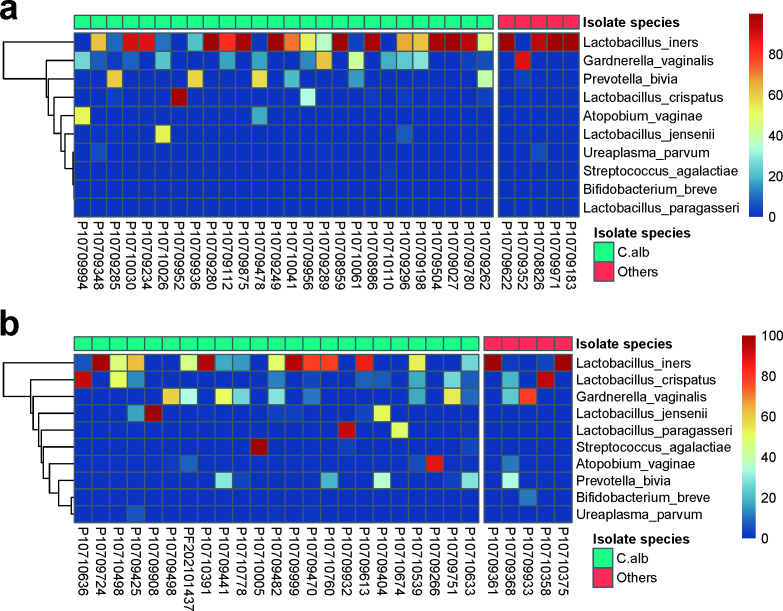
*L. iners* dominated the majority of RVVC patients with non-albicans *Candida* infection. (a) The distribution of the top 10 species of vaginal flora abundance in the RVVC group in cases of *Candida albicans* and non-albicans *Candida* infection. (b) The distribution of the top 10 species of vaginal flora abundance in the VVC group in cases of *Candida albicans* and non-albicans *Candida* infection. The heatmap shows the abundance proportion of each species, and the upper legend shows the information of *Candida* species.

The results of fungal cultivation showed that each patient enrolled in this study had only one species of *Candida* isolate. Among the 31 RVVC patients, 26 strains of *Candida albicans* (83.87%) and 5 strains of non-albicans *Candida* (NAC) (16.13%) were cultured. The NAC isolates included four *Candida glabrata* and one *Candida krusei*. In the 28 VVC patients, 23 strains of *C. albicans* (82.14%) and 5 strains of NAC (17.85%) were cultured, with the NAC consisting of three *Candida glabrata*, one *Candida tropicalis*, and one *Candida parapsilosis*. A χ^2^ test indicated no significant difference in *Candida* species between the VVC and RVVC groups (*P* = 1.0). To examine whether there were differences in *Candida* species between VVC and RVVC patients, and to explore their relationship with the microbiota, a correlation analysis was conducted. The results showed no significant association between the *Candida* species and microbiota in both the RVVC and VVC groups. However, the distribution characteristics revealed that *L. iners* was predominant in NAC-infected patients, particularly in the NAC-infected RVVC patients (four out of five) (Fig. 3). Our observations suggest an interesting trend where *L. iners* is more commonly found in NAC-infected RVVC patients, though further studies with larger data sets are needed to examine this potential linkage.

### RVVC patients have a high α-diversity of vaginal microbiota, and a high proportion of samples dominated by *L. iners*

**Fig 4:**
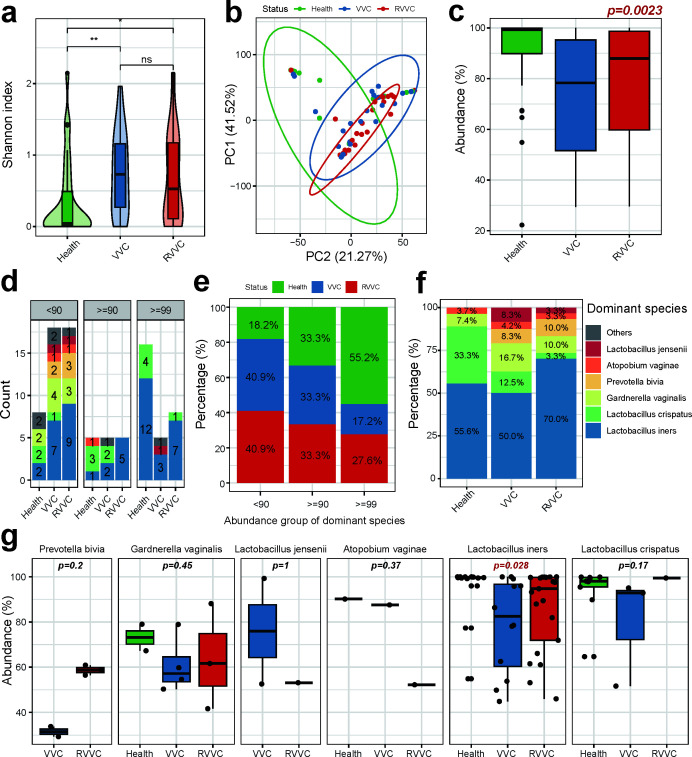
RVVC and VVC patients have a vaginal microbiota with higher diversity, and higher prevalence of *L. iners*-dominated samples shown in RVVC patients. (a) α-Diversity (Shannon index) of vaginal microbiota in the RVVC group, VVC group, and healthy group. α-Diversity of vaginal microbiota in the healthy group was significantly lower than that in the VVC and RVVC groups. (b) PCA of the β-diversity of vaginal microbiota. The first principal component explaining 41.52% of the variation and the second principal component explaining 21.27% of the variation. (c) The difference of abundance of dominant species of vaginal microbiota. (d and e) The sample size and proportion of the healthy group, VVC group, and RVVC group were shown for different ADS. (f) The distribution of the dominant species in the healthy group, RVVC group, and the VVC group. (g) The sample of the *Lactobacillus iners* was the most in the RVVC group, and the abundance was significantly higher than the healthy group and the VVC group (*P* = 0.028). Samples of *Prevotella bivia* and *Gardnerella vaginalis* were only shown in the VVC group and the RVVC group. The *Lactobacillus crispatus* in the healthy group was higher than the VVC group and the RVVC group, and the difference was not statistically significant (*P* = 0.17). Statistical significance was calculated using Wilcoxon signed-rank test.

The microbial α-diversity was calculated based on operational taxonomic units, using the Shannon-Wiener index values. The results demonstrated that the α-diversity of vaginal microbiota in the healthy group was significantly lower than that of the VVC group and RVVC group (*P* = 0.0052 and *P* = 0.012, respectively). However, there was no significant difference in α-diversity between the VVC group and RVVC group (*P* = 0.67) (Fig. 4a). Principal component analysis (PCA) was employed to assess β-diversity of vaginal microbiota, with the first principal component explaining 41.52% of the variation and the second principal component explaining 21.27% of the variation. The results indicated that the samples from the RVVC group exhibited greater clustering, whereas there was greater within-group variation presented in the VVC group and the healthy group (Fig. 4b).

Due to limited diversity of vaginal microbiota, most samples were dominated by a single species, such as the dominant species those represented in each CST, including *L. crispatus, L. gasseri*, *L. iners*, *L. jensenii*, and *G. vaginalis*. For some samples, a single species could exceed 90% of the total microbiota species. Dominant species analysis revealed that the healthy group had a significantly higher abundance of dominant species compared to the VVC and RVVC groups, suggesting a more homogenous vaginal microbiota in healthy individuals (Fig. 4c). Further classification of samples was conducted based on the ADS as ADS < 90%, 99% < ADS ≤ 90%, and ADS ≥ 99%. Among ADS ≥ 99% samples, the healthy group had a proportion exceeding 50%, with the *Lactobacillus* genus being the predominant group. In the case of 99% < ADS ≤ 90%, the sample numbers were consistent across all three groups. When ADS < 90%, a majority of the samples originated from the VVC and RVVC groups, and the dominant species came from multiple genera (Fig. 4d and e). These results indicate a higher ADS in the healthy group, suggesting single-species dominance may infer a healthy state. In contrast, the lower ADS in the VVC and RVVC groups implies weaker competitive power of dominance species in microbiota, resulting in a more complex and unstable microbial structure. Comparatively, the proportion of *L. crispatus*-dominated individuals was higher in the healthy group (33.3%), with the lowest in the RVVC group (3.3%), suggesting an important role for *L. crispatus* in protecting the vaginal environment against *Candida* infection (Fig. 4f). It is worth noting that *L. iners* dominated the microbiota in as much as 70% of individuals in the RVVC group, with a significantly higher abundance compared to the VVC group (*P* = 0.028). This suggests that VVC recurrence may be more likely when *L. iners* is the dominant species in vaginal microbiota (Fig. 4f and g). Additionally, samples dominated by *G. vaginalis* had the highest proportion in VVC (16.7%), while the proportions in RVVC (10.0%) and healthy groups (12.1%) were similar, and showed no significant difference in abundance between the three groups (*P* = 0.45), indicating that the vaginal microbiota dominated by *G. vaginalis* is more prone to VVC infection, but not the main cause of recurrence (Fig. 4f and g). Moreover, samples dominated by *P. bivia* and *L. jensenii* only appeared in the VVC and RVVC groups, with higher abundance of *P. bivia* in the RVVC group and higher abundance of *L. jensenii* in the VVC group (Fig. 4g).

### The carbohydrate metabolism and peptidoglycan synthesis pathways of the vaginal microbiota are associated with the onset and recurrence of VVC

**Fig 5:**
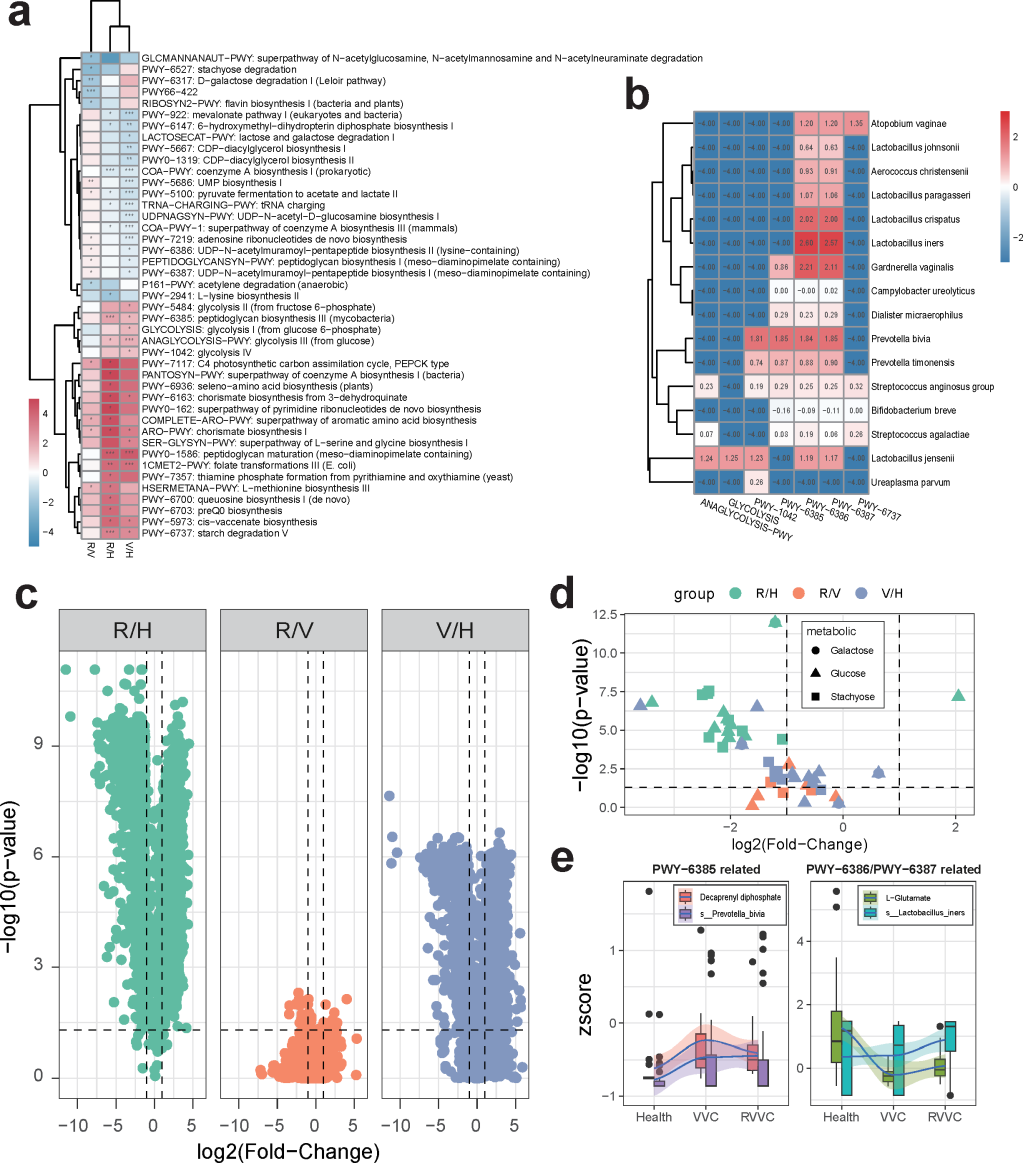
The carbohydrate metabolism and peptidoglycan synthesis pathways of the vaginal microbiota are associated with the onset and recurrence of VVC. (a) The pathway pairwise comparison among the RVVC, VVC, and healthy groups. (b) Heatmap of the contribution of a species to the differential pathways involved in carbohydrate metabolism and peptidoglycan synthesis in a microbiome, with the numbers in the squares representing the relative abundance of that species’ contribution. (c) The metabolite pairwise comparison among the RVVC, VVC, and healthy groups. (d) Volcanic map of metabolite involved in the metabolism of galactose, glucose, and hydrothreose in the RVVC group, VVC group and healthy group. The red dots represent the results of RVVC compared to the VVC group, the green dots represent the results of RVVC compared to the healthy group, and the blue dots represent the results of VVC compared to the healthy group. The metabolites on the left and right sides of the vertical dashed line are significantly upregulated or downregulated, and those above the horizontal dashed line indicate that the difference is significant. (e) Correlation analysis of differential metabolites decaprenyl diphosphate and *P. bivia* with L-glutamate and *L. iners*. Statistical significance was calculated using Wilcoxon signed-rank test. (*, *P* < 0.05; **, *P* < 0.01)

We further performed differential analysis of enriched metabolic pathways based on metagenomic data of vaginal microbiota among the healthy, VVC, and RVVC groups. The results showed that pathways involved in various carbohydrate metabolism processes were significantly upregulated in both the VVC and RVVC groups compared to the healthy group. This includes ANAGLYCOLYSIS-PWY: Glycolysis (from glucose), PWY-6737: Starch degradation, and glycolytic pathways I, II, and IV (GLYCOLYSIS, PWY-5484, PWY-1042), which were also significantly upregulated in the VVC group. Additionally, the peptidoglycan synthesis pathway PWY-6385 and the peptidoglycan maturation pathway PWY0-1586 were significantly upregulated in both the VVC and RVVC groups, indicating an increased risk of *Candida* infection. When comparing the RVVC and VVC groups, we found that the pathways involved in the synthesis of peptidoglycan precursors, PWY-6386: UDP-MurNAc-pentapeptide biosynthesis (including lysine), and PWY-6387: UDP-MurNAc-pentapeptide biosynthesis (including diaminopimelic acid) were significantly upregulated in the RVVC group, suggesting a potential association with VVC recurrence (Fig. 5a). Furthermore, *P. bivia* had the highest contribution to PWY-6385, and *L. iners* had the highest contribution to PWY-6386 and PWY-6387. *P. bivia*, *A. vaginae*, and *L. jensenii* had the highest contribution to glycolysis-related pathways (Fig. 5b).

Metabolomics analysis on vaginal discharge was also conducted. A total of 18,268 metabolites were detected, and both the RVVC and VVC groups showed significant differences compared to the healthy group, with 12,007 and 10,106 differential metabolites, respectively. Particularly in the RVVC group, the differential metabolites accounted for 65.7% of the total metabolites (Fig. 5c). This result indicates a significant impact of *Candida* infection on the metabolic environment of the vagina, and suggests a close association between VVC recurrence and the metabolic microenvironment. In addition, we performed pathway analysis on the differential metabolites and found that many metabolites were involved in glycogen metabolism. Among them, three glycogen species—lactose, glucose, and sucrose—showed the most significant enrichment. Distribution analysis of these three glycogen-related metabolites among the three groups revealed that the RVVC group had a significant increase in most metabolites, followed by the VVC group, while only one differential metabolite (glucose) was shared between the RVVC and VVC groups (Fig. 5d). This indicates that increased glycogen metabolism may play an important role in the vaginal environment of RVVC and VVC patients.

Based on the metabolomics data, we analyzed the metabolites associated with the differential peptidoglycan metabolic pathways PWY-6385, PWY-6386, and PWY-6387 within the vaginal microbiota. We found that the substrate of peptidoglycan synthesis in PWY-6385, decaprenyl diphosphate (DDP), was significantly upregulated in both the RVVC and VVC groups compared to the healthy group. However, there was no significant difference between the RVVC and VVC groups. Through correlation analysis with *P. bivia*, which had the highest contribution to PWY-6385, an increase was observed of *P. bivia* from healthy to VVC/RVVC (Fig. 5e). Furthermore, the intermediate metabolite L-glutamate in PWY-6386 and PWY-6387 showed significantly higher levels in the RVVC group compared to the VVC group, and the trend was consistent with changes in *L. iners* (Fig. 5e). It can be inferred that *L. iners* may contribute to increased synthesis of peptidoglycan by regulating the levels of L-glutamate, which could contribute to the recurrence of VVC.

## DISCUSSION

*C. albicans* is a commensal fungus commonly found in the female vaginal tract, inhabiting approximately 10%–20% of non-pregnant women and 30% of pregnant women. The yeast form of *C. albicans* is normally non-pathogenic to the host. However, when the host’s systemic and local immune defenses are compromised, *C. albicans* can transition from the yeast form to the hyphal form, proliferate rapidly, and invade the vaginal epithelial tissue, leading to VVC, a common inflammatory condition of the external genitalia and vaginal mucosa.

Changes in vaginal microbiota play a crucial role in the development of female reproductive tract diseases, including VVC. Li et al. (19) found a gradual depletion of *Lactobacillus* species, and a corresponding increase in *Gardnerella*, *Prevotella*, *Megasphaera*, *Roseburia*, and *Atopobium*, all of which are significantly associated with *Candida* infection (*P* < 0.001). Ceccarani et al. (4) observed that the healthy women had a predominance of *L. crispatus* in their microbiota, while a significant lower abundance of *L. crispatus* showed in the VVC group. Another study utilizing barcoded Illumina paired-end sequencing examined the vaginal discharge of five VVC patients, five RVVC patients, and two healthy women of reproductive age. The results showed that healthy women had a predominance of *Lactobacillus*, with an abundance of over 95%. Specifically, *L. crispatus* was identified as the dominant species. In VVC patients, the composition of the vaginal microbial community became more diverse, and the abundance of *Lactobacillus* decreased significantly from 68% to 0.2%. RVVC patients had a predominance of *L. iners*, accounting for approximately 85%. The study also demonstrated that the proportion of samples dominated by *Lactobacillus* in the healthy group was much higher than in the VVC and RVVC groups, while samples dominated by *Gardnerella* were highest in the VVC group (20). Similarly, our study also revealed decreased abundance of *Lactobacillus* at the genus level in VVC/RVVC patients and an increased abundance of BV-associated bacteria such as *Gardnerella*, *Prevotella*, and *Atopobium*. At the species level, there was a significant increase in the abundance of *A. vaginae* in the VVC group compared to the healthy group. Previous studies have shown increased α-diversity of vaginal microbiota in VVC/RVVC patients, and our study yielded similar results. The analysis revealed that more than half of the samples came from the healthy group when ADS ≥ 99%, mainly consisting of *Lactobacillus*. When 90% < ADS ≤ 99%, the proportions of the three groups were similar, and when ADS < 90%, most of the samples were from the VVC and RVVC groups, with dominant species derived from multiple genera. This suggests that a more diverse and unstable vaginal microbiota, with a less predominant composition, is more susceptible to *Candida* infection. In summary, the decrease in *Lactobacillus*, the overgrowth of BV-associated bacteria, the higher diversity, and the complex and unstable structure of the vaginal microbiota are all associated with VVC.

The high recurrence rate of VVC has been a significant concern in clinical practice, and recent studies have emphasized the close association between RVVC and changes in the vaginal microbiota. Interestingly, multiple research findings have indicated that RVVC patients still exhibit a dominance of *Lactobacillus* in their vaginal microbiota, unlike other populations with recurrent vaginal infections who exhibit a significant decrease in *Lactobacillus* (3, 17, 21). McKloud et al. (17) recently observed that while the abundance of *Lactobacillus* genus did not exhibit a significant change in RVVC patients, there was a noteworthy change of *Lactobacillus* species composition. The proportion of *L. crispatus* fell from 44% in healthy patients to 30% in individuals with RVVC, while *L. iners* demonstrated an increase from 19% in healthy samples to 40% in RVVC cases. Ma et al. (21) showed that the vaginal microbiota of healthy women was dominated by *Lactobacillus*, including *L. iners* and *L. crispatus*, with a small amount of *Gardnerella*, *Prevotella*, and other genera, while the vaginal microbiota of RVVC patients showed significantly decreased *L. crispatus*, with *L. iners* as the dominant genus. In our study, the abundance of *L. iners* increased significantly in the RVVC group (*P* = 0.028), while *L. crispatus* decreased. Also, the RVVC group had the lowest number of samples with *L. crispatus* dominance and the highest number of samples with *L. iners* dominance. Moreover, *L. iners* was predominantly present in NAC infectious patients, especially in RVVC patients. Previous study has shown that *L. iners* prompt, while *L. crispatus* inhibit the growth, colonization, and virulence of *Candida* species (11, 22). It has long been confirmed that *L. crispatus* plays a protective role in maintaining vaginal health by producing D-lactic acid and bacteriocins (23, 24). *L. iners* does not contribute strongly to the stability of the vaginal environment, as it produces L-lactic acid, which is insufficient to inhibit the progression of pathogens during vaginal infections (25). Moreover, certain distinctive attributes of *L. iners*, including a small genome and its ability to produce toxins, have been identified as factors that potentially break the balance of microbial communities, such as those found in cases of BV (26–28). In summary, our conclusion is consistent with some previous research findings: during VVC, there is an imbalance in the vaginal ecosystem with a decrease or change in the colonization of lactobacilli. Although lactobacilli still dominate at the genus level, changes in species-level composition, such as an increase in *L. iners* and a decrease in *L. crispatus*, may be important factors in the development of RVVC.

The metabolic products of vaginal microbiota play a crucial role in maintaining health, and variations in their composition and abundance reflect changes in the microbial community. This study found that several metabolites significantly increased in the vaginal environment of patients with VVC/RVVC, and these metabolites were primarily involved in glycogen metabolism. The three most enriched glycogen metabolites were lactose, glucose, and trehalose. Vaginal epithelial cells contain abundant glycogen, and the dissolved glycogen released by these cells is an important source of nutrition within the vaginal environment. Jenkins’ research demonstrated that certain bacteria, including *L. crispatus*, *L. iners*, *Mobiluncus mulieris*, *P. bivia*, and *G. vaginalis* all produce glycoside hydrolase type 1 glycogen branching enzyme (PulA, EEU28204.2), but exhibit different activities at pH 4.0. *L. iners* and *P. bivia* demonstrate stronger glycogen metabolism capabilities and higher metabolic activity at vaginal acidic conditions compared to *L. crispatus* (29). This increased nutrient production through glycogen metabolism which may contribute to the overgrowth of *Candida* species, leading to the onset of VVC.

Peptidoglycan is a major component of the cell wall in Gram-positive bacteria and is also present in some Gram-negative bacteria, providing structural integrity and resistance to osmotic pressures. It can activate the host’s innate immune response through the recognition by host peptidoglycan recognition proteins, and help maintain a healthy beneficial gut microbiota, protecting the host from inflammation, tissue damage, and colitis (30). However, a recent study indicated specific fragments of peptidoglycan have pro-inflammatory effects through interaction with pattern recognition receptors on host tissue cells and circulating leukocytes, and the peptidoglycan in the gut microbiota may be a driving factor in chronic brain inflammation (31). The healthy vaginal environment has been reported to have the highest capacity for peptidoglycan degradation since the dominant bacteria are Gram-positive *Lactobacillus* species. Maintaining high levels of peptidoglycan helps reduce the risk of HIV infection (32). However, no studies have yet shown whether the peptidoglycan producing ability of vaginal microbiota is associated with RVVC. This study revealed that the vaginal microbiota of VVC/RVVC patients have a higher capacity for peptidoglycan biosynthesis. DDP, an important substrate in this process, was significantly increased in VVC/RVVC patients, which is highly associated with *P. bivia*. Xu et al. (33) discovered that receptors similar to Nod1 and Nod2 in *C. albicans* can recognize peptidoglycan subunits, leading to the enhanced filamentous growth of *C. albicans* and the activation of its pathogenicity. And the positive correlations of *P. bivia* with VVC have been reported in many previous studies (34, 35). Hence, we conclude that *P. bivia* may promote peptidoglycan generation by increasing DDP, thereby leading to *Candida* infection.

Similarly, the interaction between the vaginal microenvironment and vaginal microbiota also influences the recurrence of VVC (36). L-glutamate levels in the vaginal environment of RVVC patients were significantly higher compared to VVC patients and positively correlated with *L. iners*. Additionally, synthesis of UDP-MurNAc-L-Ala-D-Glu, a precursor in peptidoglycan synthesis that involves L-glutamate, was significantly enriched in the vaginal microbiota of RVVC patients. However, there was no significant difference in downstream peptidoglycan synthesis between the two groups. This suggests that the increased abundance of *L. iners* in the vaginal environment of RVVC patients may provide more L-glutamate, leading to its accumulation due to delayed conversion into more peptidoglycan. Research has shown that under nitrogen-deprived conditions, *Candida* can utilize glutamate through nitrogen assimilation metabolism for energy production. Nitrogen metabolism processes are also involved in regulating the yeast/hyphal morphological transition of *Candida* and thereby influencing its pathogenicity (37). Based on this, we hypothesize that the enrichment of *L. iners* may influence peptidoglycan metabolism, potentially leading to the accumulation of L-glutamate and thereby promoting *Candida* hyphal growth, which could contribute to the recurrence of VVC, but further experimental validation is necessary to confirm this pathway. Considering the potential existence of non-pathogenic yeast forms of *Candida* and its symbiotic relationship with humans (38), we also cultured healthy specimens collected and did not detect any *Candida* samples. This indicates that the accumulation of L-glutamate caused by *L. iners* disrupts vaginal microbiota health only in the presence of *Candida*. This finding suggests that the coexistence of non-pathogenic yeast-like *Candida* and *L. iners* in the vaginal microenvironment may pose a higher risk of VVC infection and recurrence, warranting early intervention when necessary.

This study has limitations. One limitation of this study is the reliance on culture-based methods, which may not detect all *Candida* species present, potentially overlooking mixed infections that could be identified with more comprehensive techniques such as high-throughput sequencing. Additional *in vitro* and *in vivo* experiments are required to validate the findings and the feasibility of potential therapeutic targets identified, including *L. iners*, *P. bivia*, and L-glutamate. Moreover, future research should also include a validation cohort with a larger sample size to confirm the findings mentioned in this study.

### Conclusion

In conclusion, the susceptibility to *C. albicans* infections in individuals with a less diverse microbial structure and reduced levels of *L. crispatus* in the vaginal microbiota, along with an increase in pathogenic bacteria associated with BV, highlights the importance of maintaining a balanced vaginal microbiota. Additionally, increased glycogen in the vaginal environment may contribute to the abundance of *P. bivia* and subsequently prompt the infection of *C. albicans* by promoting the synthesis of peptidoglycan from DDP. Moreover, in cases of *C. albicans* infection, if the vaginal microbiota is dominated by *L. iners*, this will stimulate the production of peptidoglycan and result in the accumulation of L-glutamate, thereby promoting the growth of *C. albicans* hyphae and leading to recurrence of VVC. This study enhances our knowledge of the vaginal microbiota dynamics and the role of associated metabolites in individuals with VVC and RVVC. Such insights have the potential to significantly impact the management and prevention of *C. albicans* infections.

## SUPPLEMENTAL MATERIAL

Table S1 spectrum.01521-24-s0001.docx Relatively abundances of top 10 abundant species were varied among three groups.

ASM does not own the copyrights to Supplemental Material that may be linked to, or accessed through, an article. The authors have granted ASM a non-exclusive, world-wide license to publish the Supplemental Material files. Please contact the corresponding author directly for reuse.
