# Supplementary material for: Highlight signatures of vaginal microbiota and metabolome contributed to the occurrence and recurrence of vulvovaginal candidiasis
Source: Microbiol Spectr. 2024 Oct 30;12(12):e01521-24. doi: 10.1128/spectrum.01521-24 (PMC11619578; doi:10.1128/spectrum.01521-24)
Supplement: Table S1 — Relatively abundances of top 10 abundant species were varied among three groups. [file spectrum.01521-24-s0001.docx]

**Supplementary Table 1.** Relatively abundances of Top 10 abundant species were varied among three groups.

| **Taxonomy** | **Health** | **VVC** | **RVVC** |
| --- | --- | --- | --- |
| Others | 11.30 | 8.66 | 8.69 |
| *Bifidobacterium breve* | 2.11 | 0.40 | 0.00 |
| *Ureaplasma_parvum* | 2.44 | 0.52 | 0.58 |
| *Streptococcus agalactiaes* | 0.66 | 3.89 | 0.10 |
| *Lactobacillus paragasseris* | 0.58 | 5.08 | 0.01 |
| *Lactobacillus jensenii* | 0.39 | 6.47 | 2.14 |
| *Atopobium_vaginae* | 2.71 | 4.23 | 2.27 |
| *Prevotella bivia* | 0.30 | 5.66 | 8.33 |
| *Gardnerella vaginalisi* | 8.33 | 14.20 | 12.40 |
| *Lactobacillus crispatus* | 28.39 | 12.80 | 4.78 |
